# Impact of mixing insufficiencies on L-phenylalanine production with an *Escherichia coli* reporter strain in a novel two-compartment bioreactor

**DOI:** 10.1186/s12934-023-02165-4

**Published:** 2023-08-13

**Authors:** Manh Dat Hoang, Ingmar Polte, Lukas Frantzmann, Nikolas von den Eichen, Anna-Lena Heins, Dirk Weuster-Botz

**Affiliations:** Chair of Biochemical Engineering, TUM School of Engineering and Design, Technical University of Munich, Boltzmannstr. 15, 85748 Garching, Germany

**Keywords:** *Escherichia coli*, Reporter strains, Amino acid production, Two-compartment bioreactor, Coiled flow inverter, Population heterogeneity

## Abstract

**Background:**

The omnipresence of population heterogeneity in industrial bioprocesses originates from prevailing dynamic bioprocess conditions, which promote differences in the expression of cellular characteristics. Despite the awareness, the concrete consequences of this phenomenon remain poorly understood.

**Results:**

Therefore, for the first time, a L-phenylalanine overproducing *Escherichia coli* quadruple reporter strain was established for monitoring of general stress response, growth behavior, oxygen limitation and product formation of single cells based on mTagBFP2, mEmerald, CyOFP1, and mCardinal2 expression measured by flow cytometry. This strain was applied for the fed-batch production of L-phenylalanine from glycerol and ammonia in a stirred-tank bioreactor at homogeneous conditions compared to the same process in a novel two-compartment bioreactor. This two-compartment bioreactor consists of a stirred-tank bioreactor with an initial volume of 0.9 L (homogeneous zone) with a coiled flow inverter with a fixed working volume of 0.45 L as a bypass (limitation zone) operated at a mean hydraulic residence time of 102 s. The product formation was similar in both bioreactor setups with maximum L-phenylalanine concentrations of 21.1 ± 0.6 g L^−1^ demonstrating the consistency of this study’s microbial L-phenylalanine production. However, cell growth was vulnerable to repetitive exposure to the dynamically changing conditions in the two-compartment bioreactor with maximum biomass yields reduced by 21%. The functionality of reporter molecules was approved in the stirred-tank bioreactor cultivation, in which expressed fluorescence levels of all four markers were in accordance with respective process state variables. Additional evaluation of the distributions on single-cell level revealed the presence of population heterogeneity in both bioprocesses. Especially for the marker of the general stress response and the product formation, the corresponding histograms were characterized by bimodal shapes and broad distributions. These phenomena were pronounced particularly at the beginning and the end of the fed-batch process.

**Conclusions:**

The here shown findings confirm multiple reporter strains to be a noninvasive tool for monitoring cellular characteristics and identifying potential subpopulations in bioprocesses. In combination with experiments in scale-down setups, these can be utilized for a better physiological understanding of bioprocesses and support future scale-up procedures.

**Supplementary Information:**

The online version contains supplementary material available at 10.1186/s12934-023-02165-4.

## Background

The microbial production of bio-based components in large-scale bioreactors offers higher product amount in less time than small-scale counterparts but suffers from strongly fluctuating conditions due to prevailing mixing insufficiencies and mass transfer limitations [1–3]. As a result, each cell experiences various microenvironments followed by the expression of different phenotypic characteristics such as growth, stress response levels and product formation, which can lower the overall yields [4, 5]. Despite the awareness of this phenomenon, mechanistic understanding and the overall consequences on the producing strains remain uncertain [6, 7]. Recent studies showed reporter strains to be an effective approach to study single-cell physiologies and population heterogeneity in bioprocesses [8, 9]. By integration of a genetic copy of a reporter molecule adjacent to a specific gene of interest, which encodes for a specific cellular characteristic, *at-line* monitoring of single-cell physiologies and the identification of subpopulations is realized via fluorescence analysis tools such as flow cytometry. Single reporter strains have been proven to reliably reflect cellular characteristics during bioprocesses. For example, Delvigne et al. described an *E. coli* reporter strain for monitoring of the general stress response, whereas Garcia et al. established a reporter strain for detection of oxygen limitation [8, 10]. Another reporter strain was developed by Martinez et al. allowing the intracellular pH imaging in *Escherichia coli* and *Bacillus subtilis* cells [11]. While single reporter strains only enable access to one cellular characteristic, it is more efficient to engineer a multiple reporter strain as they allow the simultaneous analysis and correlation of multiple single-cell physiologies. The more reporter molecules are included in a single strain, the higher the outcome of cellular characteristics. So far, triple reporter strains have been designed that allow the reflection of the general stress response, growth behavior and oxygen limitation of single cells in bioprocesses [12, 13]. These cellular characteristics can be correlated to the single-cell productivities, which is an important parameter to consider during the scale-up of bioprocesses. By establishment of a quadruple reporter strain, such potential coherences can be revealed.

Population heterogeneity in bioprocesses is mainly investigated in scale-down bioreactors, in which dynamic conditions and process gradients present in industrial bioprocesses can be simulated at laboratory scale. One of the most popular setups is a two-compartment bioreactor using a stirred-tank bioreactor coupled to a straight continuous tubular reactor. It allows the simulation of process gradients along the tubular reactor with narrow residence time distributions under plug flow conditions with good radial mixing and poor axial dispersion [14–17]. Potential limitations arise for longer residence times, which cannot be compensated by an unlimited extension of the straight continuous tubular reactor. Especially with the current trend of miniaturization together with the rapid progress of in silico simulation of bioreactor setups and bioprocesses, the demand for compact and adjustable systems for experimental validation rises [18–21].

One promising approach is the utilization of helical tube reactors in which the tubing is coiled around a cylindrical rod. Due to the resulting centrifugal forces on the flowing fluid, secondary flow patterns emerge, leading to a characteristic double vortex circulation in the plane of the cross-section of the tube, often known as Dean vortices [22]. To circumvent fluid elements being trapped into one of these two double vortices, studies have shown the benefits of repetitive re-orientation of the Dean vortices by the introduction of 90° bends. This setup is often labelled as a coiled flow inverter in which radial mixing is enhanced while keeping the axial dispersion poor [23–25]. Hence, narrow residence time distributions can be provided even at laminar flow regimes with low volumetric flow rates. Additionally, this geometrical setup allows a compact and flexible design regarding scalability and the integration of sampling ports along the tubes. So far, coiled flow inverters have been mainly utilized in the field of flow chemistry [26, 27].

In the present study, we established a novel two-compartment bioreactor by combining a stirred-tank bioreactor with a coiled flow inverter as a bypass. While the former provides homogeneous environmental conditions with active control of temperature, pH, dissolved oxygen level and substrate feeding, the latter is a limitation zone with neither process parameter control nor the supply of oxygen or substrate. This setup was used for L-phenylalanine production with recombinant *Escherichia coli* in a fed-batch process, in which the recombinant production expression in cells was induced with IPTG for product formation [28]. In the newly engineered *Escherichia coli* FUS4 (pF81_kan, mod_) quadruple reporter strain (*E. coli* 4RP), the genes of fluorescent marker proteins were introduced to certain genetic elements to monitor the general stress response (*rpoS-mTagBFP2*), growth behavior (*rrnB-mEmerald*) and oxygen limitation (*narGHIJ-CyOFP1*). As a novelty, we introduced a further, far-red fluorescent protein mCardinal2 for monitoring of the product formation. This study aimed to investigate the impact of repetitive exposure of cells to fluctuating conditions regarding their biomass production and product formation as well as the expression of the four above mentioned fluorescent markers representing specific cellular physiologies. As a comparison, we cultivated the quadruple reporter strain in a well-mixed stirred-tank bioreactor at laboratory scale.

## Methods

### Strain engineering and cryopreservation

The host strain *Escherichia coli* FUS4 is a derivative of the *E. coli* K-12 strain with auxotrophies towards L-phenylalanine and L-tyrosine due to chromosomal deletion of the *pheA*, *aroF* and *tyrA* genes along the aromatic acid biosynthesis pathway. The transformation of the pF81_kan_ plasmid into this strain, which encodes for the *aroF*, *pheA*, *aroB* and *aroL* genes controlled by a tac promoter, allows IPTG-inducible production of L-phenylalanine. This strain was originally created and provided from the laboratory of Georg Sprenger [31]. With further integration of synthetic gene copies of the fluorescent proteins mTagBFP2, mEmerald and CyOFP1 downstream to the respective promoters of the *rpoS* gene, the *rrnB* operon or the *narGHIJ* operon, respectively, the general stress response, the growth, and the oxygen limitation of single cells were monitored as described by Hoang et al. [13]. To monitor the product formation via expression of L-phenylalanine synthesis enzymes, a synthetic copy of mCardinal2 was integrated into the pF81_kan_ plasmid downstream to the *aroL* gene but under the control of the same tac promoter. The synthetic copy of mCardinal2 was extracted and amplified from the pNCS-mCardinal2 plasmid provided by Chu et al. [32] using the primers 5’-TGTTTAACTTTAAGAAGGGGATATACATATGGTGAGCAAGGG-3’ and 5’-CTCATCCGCCAAAACAGACGAATTCATTACTTGTACAG-3’. These included an additional ribosome binding site upstream of mCardinal2 to allow expression levels of approximately 100,000 according to the UTR designer by Seo et al. [33]. Furthermore, the *aroL* gene was extracted and amplified from the pF81_kan_ plasmid with the primers 5’-AATCAGCGTAACAACAAG-3’ and 5’-GTTAAACAAATGCGCTCGAAAATC-3’ to engineer compatible ends. The three genetic fragments were joined by Gibson assembly and were transformed into electro-competent *E. coli* triple reporter (3RP) cells by electroporation. Cells of the resulting quadruple reporter strain (*E. coli* 4RP) were stored in a 20% glycerol stock at -80 °C.

### Design and key characteristics of the coiled flow inverter

**Fig. 1:**
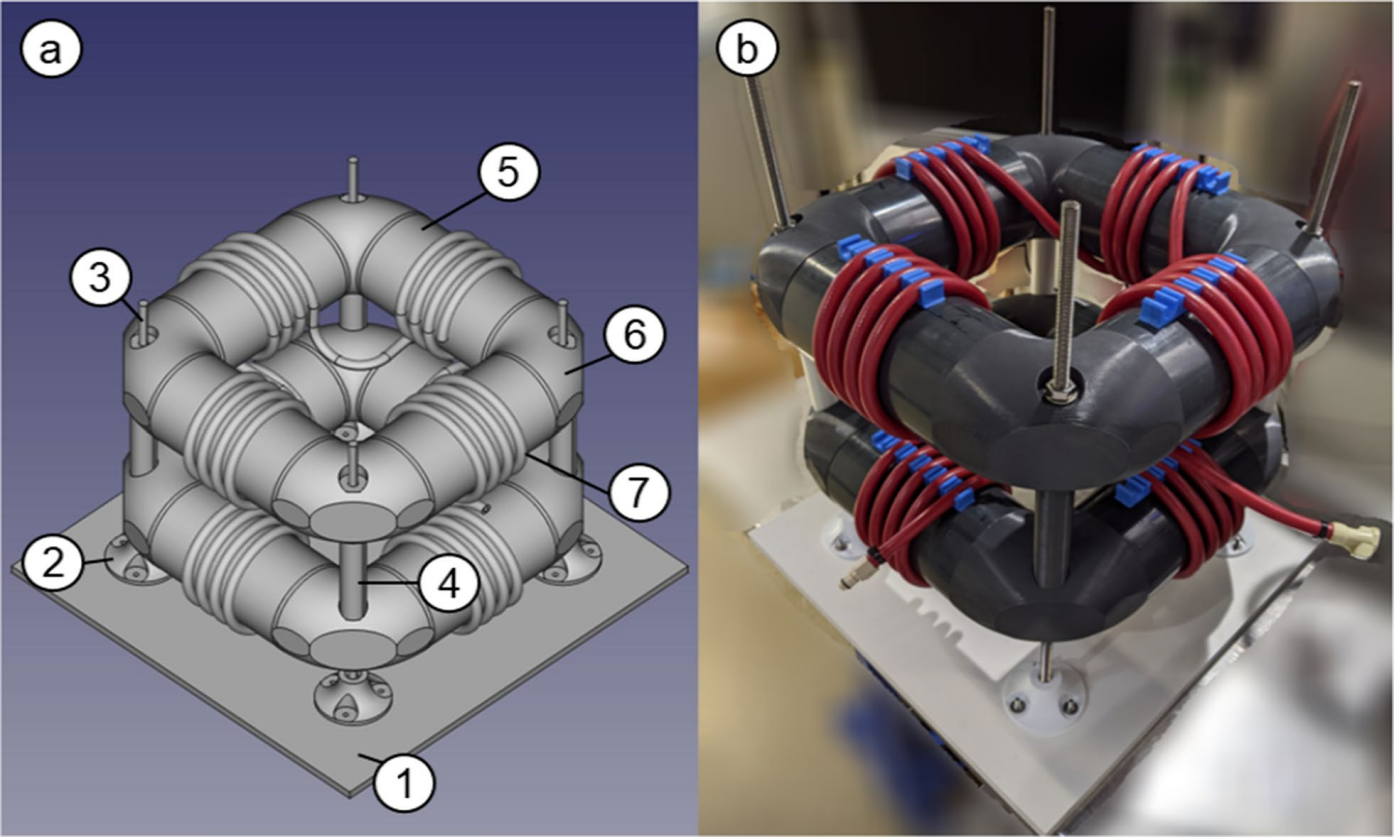
Isometric view of the constructed coiled flow inverter. **A** shows a scheme made with FreeCAD highlighting the single components used for the construction with (1) PVC plate (495 mm × 495 mm), (2) mounting foot, (3) threaded rod, (4) hollow cylinder as a spacer, (5) PVC pipe (diameter 110 mm), (6) double pipe socket with a 90° bend and (7) braided silicone tubing (inner and outer diameter of 6 and 12 mm). **B** photograph of the self-made coiled flow inverter

The coiled flow inverter (CFI) was self-constructed with commercially available components and 3D-printed parts (Fig. 1). It was built on a PVC plate (Fig. 1a (1)) as a stabilizing and carrying platform with four mounting feet (Fig. 1a (2)) attached with four threaded rods (Fig. 1a (3)). This enables the implementation of two layers with a constant distance of 120 mm by using hollow cylinders as spacer units (Fig. 1a (4)). Each layer consists of four short PVC pipes with an outer diameter of 110 mm (Fig. 1a (5)), on which hose clamp brackets are mechanically attached to ensure a fixed and reproducible coiling of the tubes. These PVC pipes are connected with 3D-printed double pipe sockets with a bend of 90° each (Fig. 1a (6)), resulting in the typical hollow square design. Braided silicone tubes with an inner tube diameter of 6 mm and an outer tube diameter of 12 mm (Fig. 1a (7)) were coiled around the PVC pipes attached to the hose clamp brackets with a coil diameter of 118 mm and a pitch distance of 20 mm. Each coiling element had 3 to 4 number of turns, after which a bend of 90° was implemented, followed by the next coiling element. Two tubes were used with a length of 6.615 m each, which corresponds to an overall volume of 373 mL. This design was based on the suggestions described by Klutz et al. [26].

### Fluid dynamic characterization of the coiled flow inverter

Mean hydraulic residence times and the axial dispersion of the coiled flow inverter were measured by the step input method using de-ionized water as a mobile phase and a vitamin B12 solution (0.025 mg mL^−1^) as a tracer solution [26, 27, 34]. For these experiments, the coiled flow inverter was connected to an ÄKTA pilot system (GE Healthcare, USA) equipped with P-907 and P-908 piston pumps and an UV cell (UV-901). The device was operated with the Unicorn software (Version 5.2). Switching between the mobile phase and the trace solution was realized by a three-way valve prior to the piston pumps. The tracer output was measured by absorbance at 280 nm with the UV cell and data acquisition intervals of 0.1—0.2 s. Based on the dispersion model, which is applicable in systems with small deviations from plug flow (e. g. turbulent regimes), raw data signals were transformed from the step input curve (F curve) to the corresponding exit age distribution (E curve) by differentiation.

1 $$E\left(t\right)=diff(F\left(t\right))$$

Consequently, the mean hydraulic residence time τ can be calculated by numerical integration of the following equation, with t being the time in s.

2 $$\tau =\int t\cdot E\left(t\right) dt$$

The dimensionless time θ and the corresponding dimensionless absorbance values F(θ) were determined by the division of each time point with the mean hydraulic residence time and by division of each absorbance value A_t_ with the maximum measured absorbance A_max_ for each run, respectively.

3 $$\theta =\frac{t}{\tau }$$

4 $$F(\theta )=\frac{{A}_{t}}{{A}_{max}}$$

Both the dimensionless absorbance F(θ) and the dimensionless time θ were plotted and the Bodenstein number Bo was calculated by a self-written MATLAB script that employs the available curve fitting toolbox of the MATLAB software (R2017b Version 9.3.0.713579) as a minimization problem with the Bodenstein number Bo as a free variable.

5 $$F(\theta )=\frac{1}{2}\cdot \left[erf\left(\frac{1}{2}\cdot \sqrt{Bo}\cdot \left(\theta -1\right)\right)+1\right]$$

This equation is applicable for narrow residence time distributions with a minimum Bodenstein number above 100 and indicates sufficient radial mixing with poor axial dispersion in tubular flow [34].

### Bioreactor cultivations

#### Pre-culture conditions

Cryopreserved cells of *E. coli* 4RP were thawed on ice for 30 min and subsequently streaked on minimal medium (5 g L^−1^ ammonium sulfate, 3 g L^−1^ monopotassium phosphate, 12 g L^−1^ dipotassium phosphate, 0.1 g L^−1^ sodium chloride, 0.015 g L^−1^ calcium chloride dihydrate, 0.1125 g L^−1^ iron(II)phosphate heptahydrate, 1.5 g L^−1^ tri-sodium citrate dihydrate, 0.3 g L^−1^ magnesium sulfate heptahydrate, 0.0075 g L^−1^ thiamin hydrochloride, 0.075 g L^−1^ L-phenylalanine, 0.075 g L^−1^ L-tyrosine, 0.05 g L^−1^ kanamycin, pH 7 with 3 M HCl) agar plates (20 g L^−1^ agar) enriched with 7 g L^−1^ glycerol. After incubation for at least 66 h at 37 °C, single colonies were picked for inoculation of 100 mL shake flasks with 10 mL of minimal medium enriched with 7 g L^−1^ glycerol and were cultivated at 150 rpm (excentricity of 5 cm) and 37 °C in an orbital shaker (Multitron, Infors HT, Switzerland) for a minimum of 24 h. The optical density at 600 nm (OD_600_) was measured (Genesys 10UV, Thermo Fisher Scientific, USA) to inoculate 500 mL shake flasks with 100 mL minimal medium enriched with 7 g L^−1^ glycerol and a starting OD_600_ of 0.01. The cells were further incubated at 250 rpm at 37 °C until they reached the exponential growth phase after around 24 h. The cell harvest was initiated by centrifugation at 3260×*g* for 10 min at 10 °C (Rotixa 50 RS, Hettich, Germany). After discarding the supernatant, the cell pellets were suspended in fresh minimal medium and the OD_600_ was measured. The stirred-tank bioreactor cultivation was initiated with a starting OD_600_ of 0.05, whereas the bioprocess in the two-compartment bioreactor was inoculated with an initial OD_600_ of 0.06.

#### Stirred-tank bioreactor cultivation

**Fig. 2:**
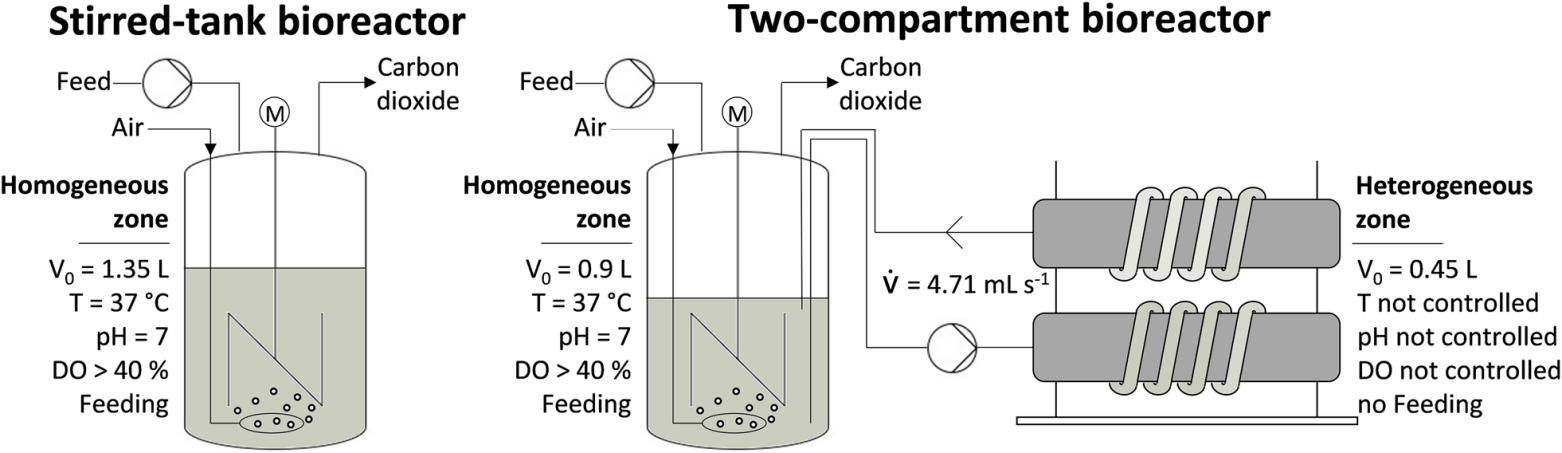
Schematic overview of the utilized bioreactor setups for L-phenylalanine production with recombinant *Escherichia coli* 4RP. The fed-batch cultivation in a stirred-tank bioreactor was characterized by consistent homogeneous conditions and the control of all process variables. In comparison, the two-compartment bioreactor setup combined a homogeneous zone in the stirred-tank bioreactor with a heterogeneous and uncontrolled zone within the coiled flow inverter with repetitive circulation of the cell suspension between both compartments applying a volumetric flow rate of 4.71 mL s^−1^, which corresponds to a mean hydraulic residence time of 102 s in the bypass

The stirred-tank bioreactor (Labfors 5, Infors AG, Switzerland, Fig. 2, left) was equipped with three equidistant baffles and three six-blade flat-blade turbines with a clearance of 38, 76 and 122 mm to the vessel bottom. A pH electrode (EasyFerm Plus PHI Arc 325, Hamilton, USA) and a dissolved oxygen level (DO) probe (VisiFerm DO Arc 325 H0, Hamilton, USA) were calibrated with a two-point (pH 4.0 and 7.0) and one-point calibration (100% DO) prior to inoculation of the bioreactor. After heat sterilization at 121 °C for 20 min with 1.35 L diH_2_O, the liquid was exchanged with the cultivation medium under sterile conditions. Here, the fed-batch process was started with an initial volume of 1.35 L of minimal medium with 4 g L^−1^ glycerol enriched with 1 mL L^−1^ of trace element solution (10 g L^−1^ aluminium chloride hexahydrate, 0.5 g L^−1^ boric acid, 7.33 g L^−1^ cobalt(II) chloride hexahydrate, 1 g L^−1^ copper(II) chloride dihydrate, 11.2 g L^−1^ manganese(II) sulfate monohydrate, 2 g L^−1^ sodium molybdate dihydrate and 2 g L^−1^ zinc sulfate heptahydrate, HCl for dissolvation). Additionally, the initial aeration rate was set to 1.35 L min^−1^. The temperature was kept at 37 °C, the pH at 7.0 by titration of either 42% phosphoric acid or 25% ammonia and the dissolved oxygen levels were kept above 40% air saturation by stepwise increase of the stirrer speed (maximum at 1500 rpm) or aeration rate (maximum at 5 L min^−1^). Overall, the bioprocess can be divided into three phases: the initial batch phase, the biomass production phase and the product formation phase. During the biomass production, an exponential feed was applied with a set growth rate of µ_set_ = 0.1 h^−1^, while a constant feeding of 0.18 g_Substrate_ g_Biomass_^−1^ h^−1^ was applied in the product formation phase. The feeding solutions one and two were applied for the biomass production phase and contained 120 g L^−1^ glycerol, 2.5 g L^−1^ L-phenylalanine, 3.6 g L^−1^ L-tyrosine, 60 g L^−1^ ammonium sulfate and 0.1 g L^−1^ kanamycin for feeding solution one, whereas feeding solution two consisted of 400 g L^−1^ glycerol, 1.11 g L^−1^ L-phenylalanine, 3.8 g L^−1^ L-tyrosine, 25 g L^−1^ ammonium sulfate and 0.1 g L^−1^ kanamycin. Both solutions were titrated with either 5 M potassium hydroxide or 25% ammonia to dissolve L-tyrosine. Minimal medium without amino acids and glycerol of 6.75 mL and 13.5 mL were added at the beginning and the middle of the biomass production phase. The induction of protein expression by supplementation with 1 mL L^−1^ IPTG (0.3 M) after reaching biomass concentrations of around 20 g L^−1^ was accompanied by the addition of 1 mL L^−1^ calcium chloride dihydrate (15 g L^−1^), 5 mL L^−1^ iron(II) sulfate heptahydrate dissolved in tri-sodium citrate dihydrate (22.5 g L^−1^ and 300 g L^−1^), 1 mL L^−1^ magnesium sulfate heptahydrate (300 g L^−1^) and 1 mL L^−1^ thiamine hydrochloride (7.5 g L^−1^) together with supplementation of feeding solution three (800 g L^−1^ glycerol, 8 g L^−1^ ammonium sulfate, 8 g L^−1^ ammonium phosphate and 0.1 g L^−1^ kanamycin, complemented with 16% (v/v) of minimal medium without amino acids and glycerol) at a constant rate of 0.18 g_Glycerol_ g_Biomass_^−1^ h^−1^. Samples were withdrawn frequently for cell dry weight measurements, high-performance liquid chromatographies (HPLC) of metabolites and flow cytometric analysis of fluorescence levels. Proportions of oxygen and carbon dioxide in the off-gas were monitored with a BlueVary gas sensor (BlueSens, Germany). The oxygen transfer rates (OTR) and carbon dioxide transfer rates (CTR) were calculated with the following equations (V̇_g,in_—inlet gas flow rate (L h^−1^), V_L_—molar volume of ideal gas (22.4 L mol^−1^), V_R_—bioreactor volume (L), Y_O2,in_—molar fraction of oxygen in inlet flow (0.2095), Y_CO2,in_—molar fraction of carbon dioxide in inlet flow (0.0004), Y_O2,out_—molar fraction of oxygen in exhaust (-), Y_CO2,out_—molar fraction of carbon dioxide in exhaust(-)).

6 $$OTR=\frac{{\dot{V}}_{g, in}}{{V}_{L}\cdot {V}_{R}}\cdot \left({Y}_{O2, in}-\frac{1-{Y}_{O2,in}-{Y}_{CO2, in}}{1-{Y}_{O2, out}-{Y}_{CO2,out}}\cdot {Y}_{O2, out}\right)$$

7 $$CTR=\frac{{\dot{V}}_{g, in}}{{V}_{L}\cdot {V}_{R}}\cdot \left({Y}_{CO2, out}-\frac{1-{Y}_{O2,in}-{Y}_{CO2, in}}{1-{Y}_{O2, out}-{Y}_{CO2,out}}\cdot {Y}_{CO2, in}\right)$$

#### Two-compartment bioreactor cultivation

As a preparation for the two-compartment bioreactor cultivation (Fig. 2, right), the blanking plug at the vessel top plate was exchanged with a second dip tube. Both dip tubes were mounted and adjusted in height and positioning within the fermentation broth of the stirred-tank bioreactor to have the longest distance possible from each other. Additionally, only two six-blade flat-blade turbines were integrated (clearance of 38 and 76 mm to vessel bottom). Both compartments were connected using silicone tubing with an inner and outer diameter of 4 and 8 mm, respectively. Fluid pumping was realized by a peristaltic pump (Masterflex® L/S® Digital miniflex®, Avantor, USA) with a double pumping tube (Masterflex® Norprene L/S® 16, Avantor, USA). The initial volume ratio was set to 0.9 L in the stirred-tank bioreactor and 0.45 L in the coiled flow inverter, including the volume of all connecting tubes (approximately 0.077 L). While the stirred-tank bioreactor was heat autoclaved accordingly to Hoang et al. [13], the two 6.615 m braided silicone tubes for the coiled flow inverter were dived into an autoclavable box with a volume of approximately 10 L and heat autoclaved for 30 min at 121 °C. The residual water inside the tubes was removed by sterile aeration with compressed air. The overall process strategy for the fed-batch production of L-phenylalanine with *E. coli* 4RP was identical as applied for the stirred-tank bioreactor cultivation, but with a reduced initial aeration rate of 0.9 L min^−1^. The volumetric flow rate in the bypass was set to 4.71 mL s^−1^ from the beginning of the bioprocess, which corresponds to a constant mean hydraulic residence time of 102 s in the coiled flow inverter. Thus, it would theoretically take 285 s to 434 s (beginning and end of the process) to pump the complete fermentation broth through the bypass. Samples were frequently withdrawn from a sampling port right after the outlet of the stirred-tank bioreactor. The same sample analysis was conducted as described for the stirred-tank bioreactor cultivation.

### Sample analysis

Optical densities of cell samples were measured at a wavelength of 600 nm with a Genesys 10S photometer (Thermo Fisher Scientific, USA). If necessary, samples were diluted with a phosphate-buffered saline (1 × PBS, 0.2 g L^−1^ potassium chloride, 0.24 g L^−1^ monopotassium phosphate, 8 g L^−1^ sodium chloride, 1.44 g L^−1^ disodium phosphate) to adjust OD_600_ of the cell sample to the linear working range of the device of 0.05 to 0.3.

Cell dry weights (CDW) were measured gravimetrically by drying empty 2 mL microcentrifuge tubes at 80 °C for at least 24 h. Subsequently, the empty tubes were cooled to room temperature and weighed with a precision balance (XA204 DeltaRange™, Mettler Toledo, USA). Tubes were filled with 2 mL of cell suspension during bioprocesses and were centrifuged at 21,130 × *g* and 4 °C for 20 min. The supernatant was either used for the preparation of HPLC samples or discarded. The remaining cell pellets in the tubes were dried at 80 °C for at least 24 h and were weighed again. The weight difference between filled and empty tubes was divided by the applied volume to receive the cell dry weights.

Quantitative amounts of the aromatic amino acids L-phenylalanine and L-tyrosine were determined by HPLC. Samples were prepared by filtration (pore size 0.2 µm) of the supernatants derived from centrifugated cell samples during bioprocesses. Based on a derivatization method, the two necessary solutions of iodoacetic acid (6.5 mg mL^−1^ in 0.04 M bicine buffer pH 10.2 adjusted with sodium hydroxide) and ortho-phtaldialdehyde (10 mg mL^−1^ in 5 mL 0.04 M bicine buffer, 5 mL methanol and 65 µL of 3-mercaptopropionic acid) were diluted 1:7 and 1:10 with 0.04 M bicine buffer, respectively. Each derivatization reaction was performed in a destination vial with 650 µL of 0.04 M bicine buffer and 8 µL of a MCP-bicine solution (1 mL of 0.04 M bicine mixed with 2.5 µL of 3-mercaptopropionic acid). The steps of the derivatization reaction are listed in the Additional file 1: Table S1. 20 µL of the derivatized sample was injected into the HPLC (Smartline HPLC, Knauer, Germany) equipped with a Gemini C18 column (150 mm x 4.6 mm x 5 µm, 110 Å, Phenomenex Ltd., Germany) and a SecurityGuard™ C18 (Phenomenexx Ltd., Germany). With a gradient profile between a monosodium phosphate solution (20 mM, pH 7.6 adjusted with sodium hydroxide) and an ultrasonicated mixture of 45% of acetonitrile, 45% of methanol and 10% of de-ionized water, the samples were pumped through the column at a flow rate of 1.0 to 1.2 mL min^−1^ at a temperature of 40 °C. A detailed description of the applied gradient profile is available in the Additional file 1: Table S2. The elution of L-phenylalanine and L-tyrosine was monitored with a fluorescence detector (RF20-A, Shimadzu, Japan) with the excitation set to 340 nm and the emission detected at 450 nm. External standards of L-phenylalanine and L-tyrosine were measured, as well. All chromatograms were analyzed with ChromGate (Version 3.1).

Filtrated samples of the supernatant were also used to quantify acetate and glycerol in the Prominence-I LC-2030C HPLC (Shimadzu, Japan). It was equipped with an Aminex HPX-87H column (300 mm x 7.8 mm, Bio-Rad Laboratories Inc., USA) together with a micro-guard column (30 mm x 4.6 mm, Bio-Rad Laboratories Inc., USA). 10 µL of sample were measured with an isocratic profile with a 5 mM sulfuric acid solution at 0.6 mL min^−1^ and 60 °C for 30 min. The elution of acetate and glycerol was monitored by a refractive index detector (RID-20A, Shimadzu, Japan). Quantification of both components was enabled by measuring external standards, as well. The corresponding chromatograms were analyzed with LabSolutions (Version 5.98).

Expressed fluorescent proteins of the *Escherichia coli* 4RP were measured with the flow cytometer FACSMelody™ (Becton Dickinson, USA). This device has three lasers allowing the excitation at 405 nm (36 mW), 448 nm (16 mW) and 640 nm (36 mW) on a beam spot size of 9 ± 3 µm×67 ± 5 µm each. Additionally, the FACSMelody™ was equipped with nine detection filters and a sorting nozzle with a diameter of 100 µm. Cell samples were prepared by centrifugation at 21,130 × *g* for 3 min and the supernatant was discarded. The remaining cell pellets were resuspended and diluted with 1 × PBS to allow event rates between 500 to 1000 events per second in the flow cytometer. FACSFlow™ (Becton Dickinson, USA) was used as sheath fluid. During measurements, the threshold was set on the side scatter (SSC-W at 491 V) and technical triplicates of 100,000 events were recorded for every sample. The photomultiplier tubes (PMT) for forward scatter (FSC) and side scatter (SSC) were set to 225 V and 335 V, whereas detection filters at 448/45 nm for blue fluorescence, 527/32 nm for green fluorescence, 586/42 nm for orange fluorescence and 660/10 nm for red fluorescence had PMT values of 500 V, 500 V, 600 V and 600 V.

### Analysis of flow cytometry data

Recorded data of flow cytometer measurements were saved as FCS3.1 files and post-analysis of the expressed fluorescence signals of the *Escherichia coli* 4RP was executed using FCSExpress (Version 7.18.0015). Mean and median fluorescence levels, the coefficient of variances as well as standard deviations of the histograms were calculated from this software. For the later radar plots, the determined median fluorescence intensities of the whole cell population were averaged for the initial batch phase, the early and late biomass production phase (STR: 15.3 h–24.7 h and 24.8 h–38.0 h, TCB: 15.6 h–23.6 h and 23.7 h–36.7 h) as well as the early, middle and late product formation phase (STR: 38.1 h–45.9 h, 46.0 h–75.3 h and 75.4 h–89.9 h, TCB: 36.8 h–45.3 h, 45.4 h–75.4 h and 75.5 h–94.1 h) and divided by their maximum value for each detection range of each bioprocess. Stacked histograms were prepared and plotted with bi-exponential x-axis and linear y-axis, whereas density plots are shown with bi-exponential scales for both x- and y-axis. Gates were drawn manually. The level of population heterogeneity from the obtained histograms was evaluated based on their characteristics regarding the number of local maximum values, skews and the width of the distribution. The occurrence of a single maximum value with a very narrow distribution resemble a homogeneous population, whereas multiple local maximum values and a broad distribution represent high levels of population heterogeneity. Additionally, left-skewed distributions are characterized by the tendency to lean towards lower fluorescence intensities and right-skewed distributions represent the opposite trend. In both cases, these are indicators for differences on single-cell level. To support the evaluation of population heterogeneity by the observed histogram distribution values, mean-to-median ratios and coefficient of variances (CV) will be considered for all histograms. Mean-to median ratios of 1 indicate a homogeneous population based on the resulting unimodal distribution. The higher this ratio becomes, the stronger the deviation from this unimodal shape and the stronger the differences between single cells. On the other hand, CV values close to 0 represent a very narrow distribution (homogeneous population), while higher values resemble comparably broad histograms and thus higher levels of population heterogeneity [35].

## Results

### Mean hydraulic residence time and Bodenstein number in the coiled flow inverter

Prior to the establishment of the two-compartment bioreactor combining a stirred-tank bioreactor with a coiled flow inverter, the mean hydraulic residence time and the Bodenstein number in the CFI were determined by tracer pulse experiments. Measurements of triplicates at the same flow rate of 4.71 mL s^−1^ led to a mean hydraulic residence time of τ = 86.3 ± 0.6 s. Considering the additional volume of the connecting tubes between STR and CFI during TCB cultivation, mean hydraulic residence times in the bypass increased to around 102 s. Furthermore, the Bodenstein numbers were at Bo = 336.5 ± 12.6. Accordingly, corresponding residence time distributions at a volumetric flow rate of 4.71 mL s^−1^ are expected to be narrow due to good radial mixing and poor axial dispersion. Consequently, 90% of the cell population experience hydraulic residence times of 89–115 s.

### Comparison of biomass production and product formation during the L-phenylalanine production process

L-phenylalanine production processes with recombinant *Escherichia coli* 4RP were conducted in a stirred-tank bioreactor (STR) with assumed homogeneous conditions and in a novel two-compartment bioreactor (TCB) with repetitive circulation of the cultivation broth between a homogeneous zone in the stirred-tank bioreactor and the limitation zone in the coiled flow inverter.

**Fig. 3:**
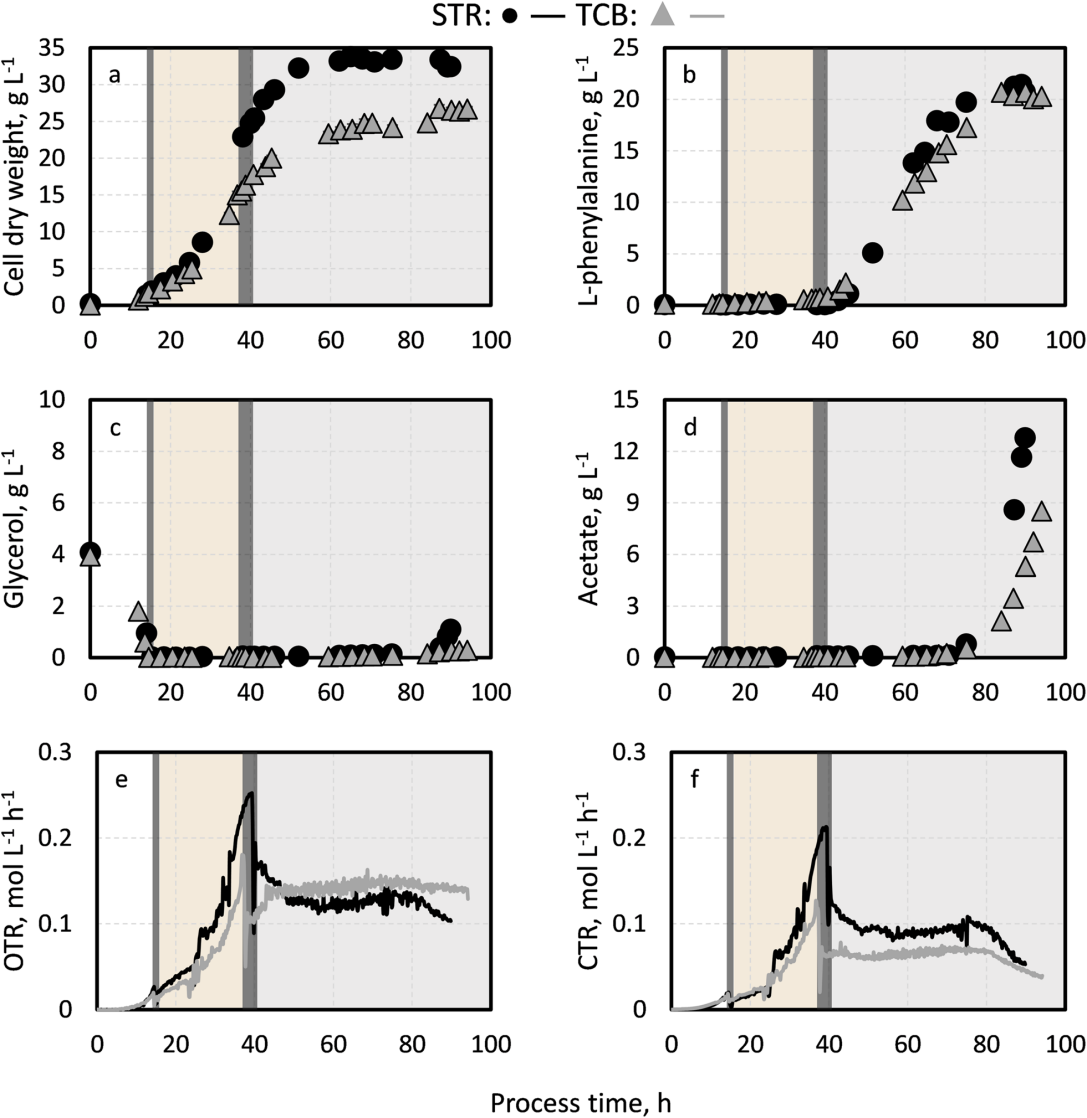
Fed-batch production of L-phenylalanine from glycerol with *E. coli* 4RP in a stirred-tank bioreactor (STR) compared to the process performance in a two-compartment bioreactor (TCB). **a**, **b** show the biomass concentration and L-phenylalanine concentration, whereas **c**, **d** depict glycerol and acetate concentration. The subplots **e**, **f** visualize the oxygen transfer rates (OTR) and carbon dioxide transfer rates (CTR). The different phases are separated by the dark grey verticals. Segments with a white background represent the initial batch phase, whereas the segments with an orange background show the biomass production phase. A grey background depicts the product formation phase. The process strategies are described in the methods section

Cell growth started in the initial batch phase (Fig. 3a), with maximum growth rates of 0.26 h^−1^ and 0.33 h^−1^ to biomass concentrations of 2.0 ± 0.2 g L^−1^ and 1.7 ± 0.0 g L^−1^ during STR and TCB cultivation, respectively. After the exponential feeding was initiated, cell growth started to differ slightly between both fed-batch processes in the early biomass production phase (Fig. 3a, orange background). The deviation became more pronounced at the late stages of this phase as biomass concentrations of 24.8 ± 0.4 g L^−1^ were reached after 39.9 h of process time in the stirred-tank bioreactor cultivation compared to 15.5 ± 0.3 g L^−1^ in the two-compartment bioreactor setup after 37.8 h. The corresponding average growth rates were at 0.12 h^−1^ and 0.10 h^−1^, respectively. Normally, the recombinant expression of L-phenylalanine synthesis enzymes was induced with 0.3 mM IPTG after surpassing a biomass target value of 20 g L^−1^, which was measured representatively through the OD_600_ (correlation factor of 0.5). However, the target value was only reached during the stirred-tank bioreactor cultivation, but not during the two-compartment bioreactor operation because dissolved oxygen levels could not be kept above 40% as both, stirrer speed and the aeration rate, reached their maximum. Consequently, the product formation phase was initiated in the TCB cultivation with a lower biomass concentration based on the measured OD_600_ values. Because of the remaining amounts of L-tyrosine in the cultivation broth (Additional file 1: Fig. S1), cells continued to grow until 62.1 h (STR) or 68.4 h (TCB) and then remained approximately level until the end of the process. Maximum biomass concentrations of 33.5 ± 0.5 g L^−1^ (STR) and 26.8 ± 0.5 g L^−1^ (TCB) were finally achieved at 75.3 h and 87.1 h. Except during the initial batch phases and at the process end, the glycerol concentrations were scarce in both processes (Fig. 3c). L-phenylalanine accumulation started after induction of the recombinant protein expression (Fig. 3b, grey background). Due to the continued cell growth, product concentrations increased linearly after depletion of the remaining L-tyrosine (Additional file 1: Fig. S1). However, it seems that the L-phenylalanine production was slightly faster in the stirred-tank bioreactor cultivation. The corresponding maximum product formation rates were at 31.5 mg g^−1^ h^−1^ in the STR, whereas cells in the TCB had maximum rates of 25.2 mg g^−1^ h^−1^. Nevertheless, maximum L-phenylalanine concentrations only differed marginally and were at 21.5 g L^−1^ (STR) and 20.6 g L^−1^ (TCB). Concomitant with the slow-down of the L-phenylalanine production at around 80 h, acetate began to accumulate in both bioprocesses (Fig. 3d, grey background).

The courses of OTR and CTR were identical in the STR and TCB cultivation (Fig. 3e, f). While both rates increased exponentially during the biomass production phases, they were constant during the product formation phases until a final drop towards the process ends. Interestingly, the proportion of carbon dioxide of the integral carbon balance was remarkably higher during the TCB cultivation in the biomass production phase with 54.6 ± 9.4 mol% compared to the STR cultivation with 47.3 ± 11.5 mol% (Table 1, Additional file 1: Tables S3 and S4).

**Table 1:** Key performance indicators during the L-phenylalanine production in a stirred-tank bioreactor (STR) or two-compartment bioreactor (TCB) with an *Escherichia coli* quadruple reporter strain

Parameter		Stirred-tank bioreactor	Two-compartment bioreactor
Integral carbon proportion of carbon dioxide during the (a) initial batch, (b) biomass production and (c) product formation phases on average, mol%	(a) (b) (c)	29.1 ± 0.0 47.3 ± 11.5 67.2 ± 0.5	27.7 ± 8.3 54.6 ± 9.4 67.4 ± 0.9
Biomass concentration at the start of product formation, g L^−1^		24.8 ± 0.4	15.5 ± 0.3
Maximum biomass concentration, g L^−1^		33.5 ± 0.5	26.8 ± 0.5
Maximum product concentration, g L^−1^		21.5	20.6
Maximum biomass specific product formation rate, mg g^−1^ h^−1^		31.5	25.2

Overall, both bioprocesses showed similar trends and process performances, but with higher maximum achieved biomass concentrations in the STR cultivation (Table 1).

### Monitoring of fluorescence expression during L-phenylalanine production at population level

**Fig. 4:**
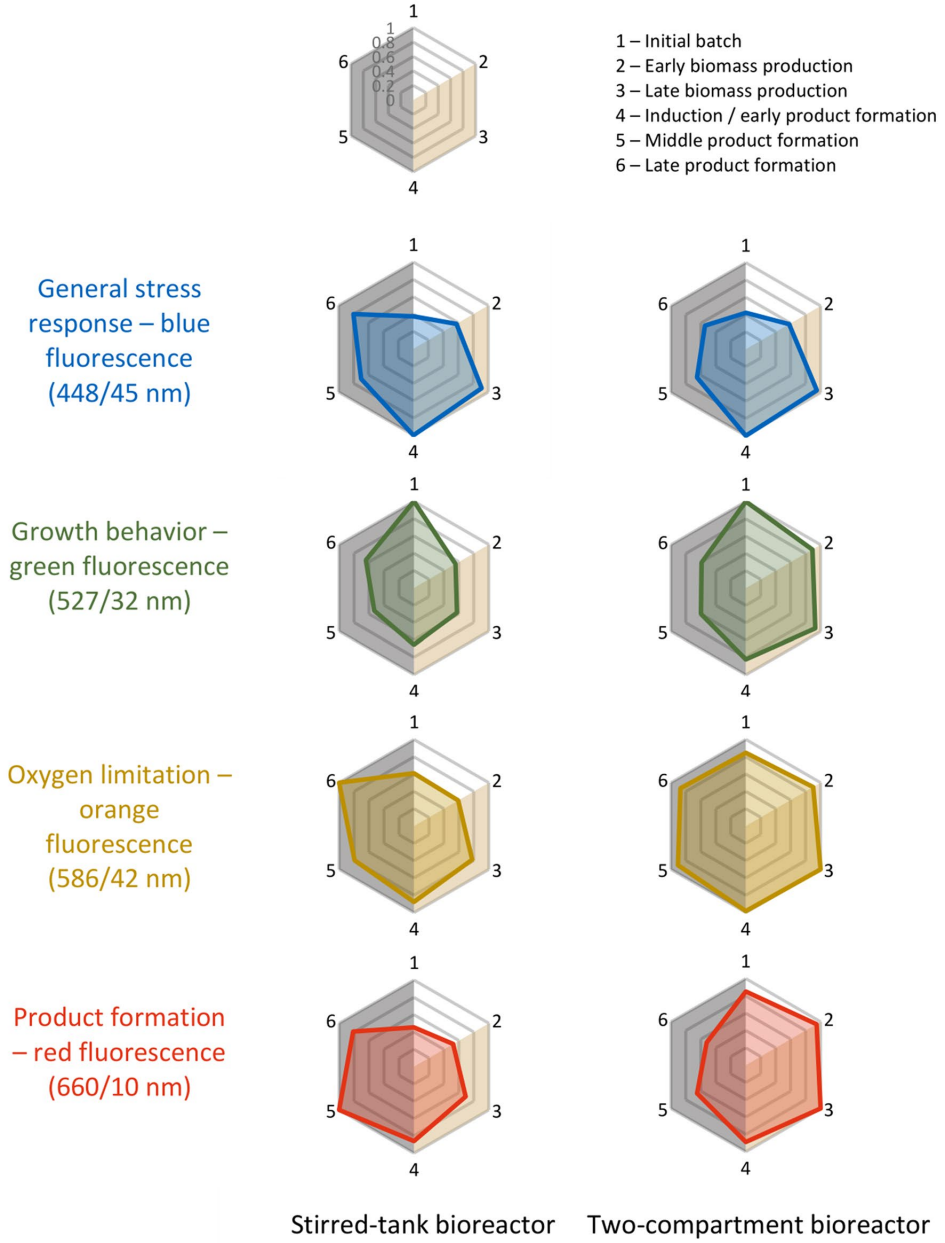
Radar plots of the normalized fluorescence intensities exhibited of the microbial population during the L-phenylalanine fed-batch production from glycerol with *E. coli* 4RP. The median fluorescence signals were averaged for the six process phases in the initial batch, early and late biomass production and the induction/early, middle and late product formation. Higher values indicate a higher expression of the corresponding cellular phenotypes in the general stress response (blue plot), growth behavior (green plot), oxygen limitation (orange plot) and product formation (red plot). The white background represents the initial batch phase, the orange background the biomass production phase and the grey background the product formation phase

Aside from the genetic modifications of the *E. coli* 4RP strain for the inducible L-phenylalanine production, *E. coli* 4RP exhibited the four fluorescent proteins mTagBFP2 (448/45 nm), mEmerald (527/32 nm), CyOFP1 (586/42 nm) and mCardinal2 (660/10 nm), whose expressions are related to the general stress response, the growth behavior, the oxygen limitation and the product formation of the cells. The highest normalized values of ‘1’ indicate the highest expressed intensities of the corresponding fluorescent proteins, which was also used as a reference value. On the other hand, lower values signalize the percentual reduction based on the reference value of monitored fluorescence intensities, e. g. ‘0.5’ corresponds to a 50% lower signal intensity and ‘0’ means a 100% reduction. As the maximum measured fluorescence intensities of each detection range differed between both processes, the general trends shall be compared (Fig. 4).

According to the expressed blue fluorescence measured at 448/45 nm during cultivation in the stirred-tank bioreactor, the general stress response levels of the cells were low at the initial batch phase (0.38) and the early biomass production phase (0.57). This increasing trend continued until the late biomass production phase and the induction of the cells, in which the general stress response levels of the cell population were the highest with values at 0.92 and 1. After induction, the expressed blue fluorescence decreased towards the end of the process. Comparably low average fluorescence intensities during the initial batch and the early biomass production phase, but with highest levels during the late biomass production phase and the induction and early product formation phase, were observed for the cell population during L-phenylalanine production in the two-compartment bioreactor, as well.

On the other hand, growth indicated by green fluorescence at 527/32 nm was highest in the initial batch phase and immediately decreased with the start of the feeding with consistent normalized values between 0.5 and 0.6 for the rest of the process. Again, cells in the two-compartment bioreactor exhibited the highest green fluorescence intensities at the late batch phase though the intensities remained at higher levels with normalized values of 0.9 during the biomass production phase. Only with the induction of the cells and the start of the product formation, fluorescence intensities at 527/32 nm further dropped to normalized values of 0.6.

In general, the oxygen limitation marker CyOFP1 (586/42 nm) is activated during oxygen limiting phases (higher fluorescence), but not with excess of dissolved oxygen within the cultivation broth (lower fluorescence). However, preliminary studies with the precursor strain showed the expression of this marker when dissolved oxygen levels came close to 40% [13]. Thus, oxygen limitation (DO at around 40% or lower) was not detected, particularly at the initial batch and early biomass production phases during the STR cultivation with normalized values of 0.55 and 0.58, respectively. Towards the end of the biomass production phase, the fluorescence intensities began to increase consistently until the end of the bioprocess, where the maximum was reached. In contrast, it seems that *E. coli* cells experienced consistent oxygen limitation during the two-compartment bioreactor cultivation according to the measured fluorescence intensities at 586/42 nm as the normalized fluorescence remained above 0.85 throughout the whole bioprocess.

Finally, the product formation of the cell population was indicated by red fluorescence based on the mCardinal2 expression, which was measured at 660/10 nm. Cells in the stirred-tank bioreactor exhibited the lowest red fluorescence during the initial batch phase, but with an increasing trend towards the end of the biomass production phase (normalized value of 0.69). The induction of the recombinant protein expression in *E. coli* 4RP cells with IPTG and the start of the product formation was accompanied by a strong rise of red fluorescence signals with its maximum at the middle of the product formation phase. Counterintuitively, the microbial population of the two-compartment bioreactor cultivation expressed the highest red fluorescence levels at 660/10 nm during the initial batch and biomass production phase, while the corresponding fluorescence intensities dropped after the initiation of the product formation phase.

### Population heterogeneity in bioprocesses at homogeneous and heterogeneous conditions

Average fluorescence intensities expressed from the *E. coli* 4RP population during L-phenylalanine production in either STR or TCB mask potential differences of exhibited single-cell fluorescences. Hence, histogram distribution values of all integrated reporter molecules shall support the identification and evaluation of possible subpopulations regarding the general stress response, growth behavior, oxygen limitation and product formation (blue, green, orange and red fluorescence) of single cells. The visual evaluation will be supported by mean-to-median ratios (MMR) and coefficient of variances (CV) (Additional file 1: Tables S5 and S6) of each histogram with higher values indicating stronger differences in single-cell fluorescence intensities.

**Fig. 5:**
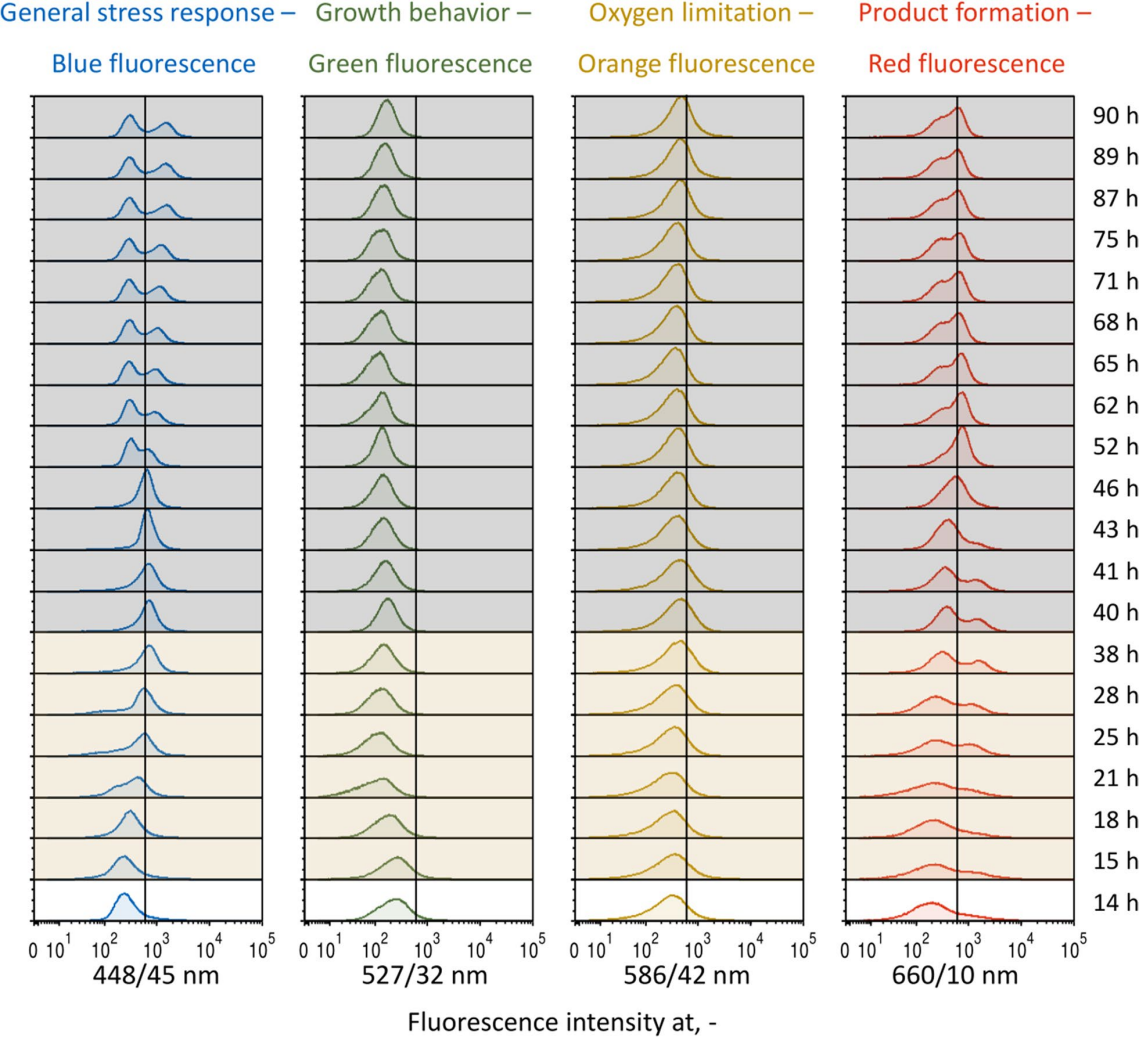
Histogram distributions of *at-line* analyzed samples during the fed-batch process for L- phenylalanine production in a stirred-tank bioreactor with *Escherichia coli* 4RP. Stacked histograms show the expressed fluorescence intensities against the relative cell count for the four markers mTagBFP2 (blue marker, general stress response), mEmerald (green marker, growth behavior), CyOFP1 (orange marker, oxygen limitation) and mCardinal2 (red marker, product formation). The corresponding time stamps of each histogram are listed on the right. Plots with a white background represent histograms during the initial batch phase, whereas histograms with an orange or grey background derive from the biomass production phase or product formation phase, respectively. The verticals in each stacked histogram layer marks a signal intensity of 6∙10^2^. The x axis is scaled bi-exponentially

According to the mean-to-median ratios and the CVs, the single-cell fluorescence of mTagBFP2 as representative of the general stress response (Fig. 5, blue subplots) differed the most at the beginning of the biomass production phase (15 h, MMR of 1.7 and CV of 2.2) and at the process end (89.9 h, MMR of 1.7 and CV of 0.9). Though the histogram of the first time point showed no remarkable deviation from a normal distribution, a stable bimodal distribution of two equally proportionated subpopulations was observed at the process end. While 54% of the cells showed a median signal intensity at 2.9∙10^2^, the other 46% of the cell population expressed higher fluorescence intensity levels at 1.3∙10^3^.

Considering the histograms of the expressed green and orange fluorescences for monitoring of growth and oxygen limitation (Fig. 5, green and orange subplots), the distributions merely changed in terms of the exhibited intensities, but did not deviate from their mono-modal shape (average MMR and CV of 1.2 ± 0.1 and 1.1 ± 0.6 at 527/32 nm and 1.2 ± 0.1 and 1.3 ± 0.4 at 586/42 nm). There was one exception for the growth marker, in which a remarkably high CV value of 3.1 was determined at 21 h. Indeed, this histogram was characterized by a left skew.

In contrary, single-cell fluorescence of mCardinal2 as a marker for the product formation (Fig. 5, red subplots) strongly differed at the initial batch and early biomass production phases as MMR and CV values of above 2.0 and 1.9 were present until 21 ﻿h. High MMR values (> 1.7) remained until 41 h of process time. These indications were underlined by the broad distributions between 14 h–21 h. Entering the late biomass production phase, distributions remained broad (standard deviation at 8.1∙10^2^), but now concomitant with two subpopulations with the smaller fraction of 19.4% showing high fluorescence intensities of 1.2∙10^3^. After a temporal fusion of the cell population to a narrow distribution (52 h, MMR of 1.1 and CV of 0.6) it seemed a second population arose towards the process end with 44% of the cells exhibited lower red fluorescence of 2.2∙10^2^ and 56% of the cells at higher levels of 5.9∙10^2^. However, the MMR and CV values did not indicate this (Additional file 1: Table S5).

**Fig. 6:**
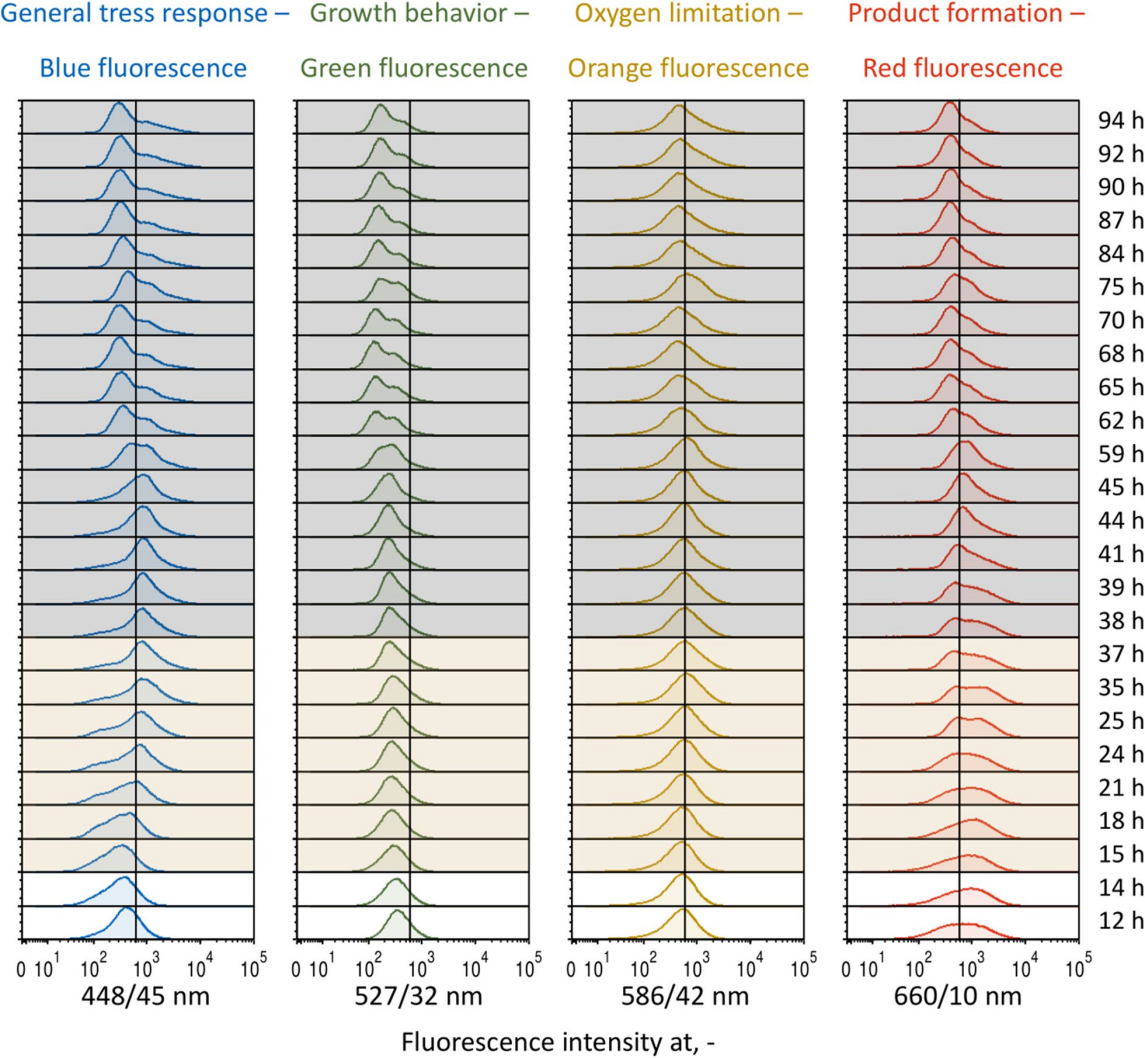
Histogram distributions of *at-line* analyzed samples during the fed-batch process for L-phenylalanine production in a two-compartment bioreactor with stirred-tank bioreactor and coiled flow inverter with *Escherichia coli* 4RP. Stacked histograms show the expressed fluorescence intensities against the relative cell count for the four markers mTagBFP2 (blue marker, general stress response), mEmerald (green marker, growth behavior), CyOFP1 (orange marker, oxygen limitation) and mCardinal2 (red marker, product formation). The corresponding time stamps of each histogram are listed on the right. Plots with a white background represent histograms during the initial batch phase, whereas histograms with an orange or grey background derive from the biomass production phase or product formation phase, respectively. The verticals in each stacked histogram layer marks a signal intensity of 6∙10^2^.The x axis is scaled bi-exponentially

For the two-compartment bioreactor cultivation, the strongest differences in the general stress response of single cells occurred at the middle of the product formation phase (62 h) and beyond. In fact, the MMR and CV values increased from 1.3 ± 0.1 and 1.0 ± 0.1 during the first 60 h of process time to over 2.0 and 1.4 at the process end (87 h–94 h). Indeed, the histograms showed right-skewed distributions starting from 62 h with 72.6% of the *E. coli* cells with lower blue fluorescence (median value of 4.3∙10^2^), whereas 28.2% had elevated fluorescence intensity levels at around 1.3∙10.^3^ (Fig. 6, blue subplots). These became even more pronounced towards the process end.

Interestingly, the same trend was valid for the corresponding single-cell fluorescences at 527/32 nm for monitoring of the growth behavior (Fig. 6, green subplots). While unimodal histograms were present during the initial batch and biomass production phases (MMR values of 1.2 ± 0.0), right-skewed distributions were observed at 84 h and later with MMR values above 1.4 (Fig. 6, green subplots).

According to the histograms addressing the oxygen limitation of the cells (Fig. 6, orange subplots), comparably wide but unimodal shapes resulted with stronger spreads towards the end of the process. This was accompanied by a consistent increase of MMR and CV values from 1.2 to 1.6 and 0.8 to 1.2, respectively.

Similar spreads of the histograms were observed for the product formation marker during the initial batch phase and the biomass production phase (Fig. 6, red subplots) with an average MMR and CV value of 1.5 and 1.0. These transformed slowly to more narrow shapes with the start of the late biomass production phase and beyond. At the end, both indicators decreased to 1.3 and 0.8.

## Discussion

### Implementation of a coiled flow inverter in a two-compartment bioreactor

Coiled flow inverters have already been established and extensively studied for flow chemistry applications. Assuming a small extent of dispersion if Bodenstein numbers are above 100 [34], a narrow residence time distribution was approved for a volumetric flow rate of 4.71 mL s^−1^. The same findings in similar coiled flow inverters were frequently reported by others [22–27]. For the integration into a novel two-compartment bioreactor, we targeted the resulting mean hydraulic residence time in the bypass to around 102 s as this corresponds to expected mixing times of 10–250 s in industrial bioprocesses [29]. Furthermore, Simen et al. (2017) reported the cellular response time of *E. coli* to fluctuating conditions to around 70 s [30].

### Process performance during L-phenylalanine production with a new *Escherichia coli* quadruple reporter strain in a novel two-compartment bioreactor

The genetic integration of synthetic copies of four reporter molecules and the corresponding expression during bioprocesses potentially damage the cellular integrity and elevate the metabolic burden [36, 37]. In this study, L-phenylalanine production from glycerol in a stirred-tank bioreactor at laboratory scale with the here established *E. coli* quadruple reporter strain led to a maximum biomass concentration of 33.8 g L^−1^ and a maximum product amount of 21.5 g L^−1^ with a maximum product formation rate of 31.5 mg g^−1^ h^−1^. In comparison, Weiner et al. [28] conducted the reference process with the original strain *E. coli* FUS4 (pF81_kan_) without fluorescent proteins in a stirred-tank bioreactor at pilot scale (10 times higher initial working volume) and achieved maximum biomass and product concentrations of 31.0 g L^−1^ and 22.8 g L^−1^ with a maximum product formation rate of 34 mg g^−1^ h^−1^ [28]. Hence, process performances with the genetically modified *E. coli* 4RP were similar to the cultivations with the original strain. This suggests no harmful effects based on the genetic modifications and the corresponding biological reactions and rules out the occurrence of metabolic burden for the cells.

In contrary, the cultivation in the TCB showed that cell growth was remarkably affected as the maximum achieved biomass concentration dropped to 26.8 g L^−1^, which means a loss of 21% compared to the cultivation in a stirred-tank bioreactor. One possible reason for the lower cell growth is the shift of carbon fluxes in the metabolism of the cells as a response to dynamic environments, which was also reported by Velastegui et al. [38]. In the here conducted L-phenylalanine production processes, carbon dioxide covered 25% more of the integral carbon balance, especially during the initial biomass production phase compared to the cultivation in the STR. Interestingly, higher carbon dioxide formation was accompanied by slightly elevated L-phenylalanine concentrations of around 0.5 g L^−1^ during the biomass production phase in the TCB, thus pointing towards a slower uptake of this essential amino acid (Additional file 1: Tables S4 and S5). The observed lower growth might have been further promoted by potential anoxic conditions within the bypass as lower biomass yields with glycerol were reported under such circumstances [39]. Although not measured, the effects of expectably lower temperatures in the bypass might have contributed to the slower increase of biomass, as well [40]. Conversely, the L-phenylalanine production was not affected as the maximum achieved concentrations in the TCB cultivation was at 20.7 g L^−1^, which is only 4% lower than the maximum achieved L-phenylalanine concentration in the STR. Though the aromatic amino acid biosynthesis is described to depend on the presence of dissolved oxygen [41], short exposure times to anoxic conditions seemed to be tolerated by the producing cells. Interestingly, the by-product formation during cultivation in the TCB was not elevated in comparison to the L-phenylalanine production in the STR.

Overall, we successfully demonstrated the L-phenylalanine production in our novel two-compartment bioreactor combining a stirred-tank bioreactor with a coiled flow inverter as a bypass. To our best knowledge, coiled flow inverters were never used in any scale-down setups. However, we foresee a great potential for CFIs in future scale-down systems and a considerable alternative to straight continuous tubular reactors, as good radial mixing and poor axial dispersion can be provided even at lower volumetric flow rates. Indeed, the here shown two-compartment bioreactor setup is not perfect yet. Particularly strategies for temperature control of the bypass and sensors for temperature, DO and/or pH monitoring within the coiled flow inverter shall be implemented to elucidate the prevailing dynamic conditions. Future studies could also investigate the effects of longer exposure times of the cells to limiting conditions regarding the cell growth and productivity of the bioprocess.

### Reporter molecules for *at-line* monitoring of expressed cellular phenotypes at population level

General trends of measured fluorescence intensities for the general stress response, growth behavior and oxygen limitation during the stirred-tank bioreactor cultivation with the *E. coli* 4RP were consistent with the achieved data of the precursor *E. coli* triple reporter strain, thus indicating the reliability and reproducibility to reflect these cellular characteristics in well-mixed bioprocesses [13].

For the novel product formation marker, red fluorescence intensities expressed from the population should be highest during the product formation phase, which was indeed the case. However, cells were already exhibiting red fluorescence before induction of the cells, underlining the basal expression of mCardinal2 in some cells. This assumption is supported by the corresponding histograms of this marker characterized by a wide spread of single-cell fluorescence at the early bioprocess. Unfortunately, tac promoters often suffer from leakage during recombinant expression, which was reported by others [42, 43]. Nevertheless, it is important to stress out that induction of the recombinant protein expression in cells with IPTG increased red fluorescence intensities stronger in a shorter time (slope of signal intensity in the early product formation phase at 20.9 h^−1^) than the basal expression in the biomass production phase (slope at 8.7 h^−1^). Hence, the product formation marker can be considered as functional, but potentially needs an optimized design to circumvent the observed basal expression.

Under dynamic environmental conditions, the general trends for the general stress response and growth behavior were approximately the same compared to the STR cultivation, but strong differences were observed for the oxygen limitation and product formation markers. While the former was anticipated due to the permanent exposure of cells to the non-aerated bypass, cells showed comparably low signal intensities for the latter after initiation of the product formation. This outcome was opposite to our expectations and is even more surprising when considering that the product formation in the TCB cultivation was similar to the STR cultivation based on the achieved L-phenylalanine concentrations.

Here, dissolved oxygen levels during cultivation in the two-compartment bioreactor might have been the decisive factor for the here occurring trends as the access to molecular oxygen is mandatory for the maturation of fluorescent proteins [44, 45]. With the assumption that anoxic conditions occurred within the bypass at higher biomass concentrations, the maturation of fluorescent proteins might have been increasingly disturbed during the product formation phase. While this was masked for the general stress response and growth markers due to the anyway low expression levels towards the process end, especially the product formation marker seemed to be affected by this.

Detailed insights of in vivo maturation and decay rates for the here used reporter molecules in *Escherichia coli* are mandatory for a better understanding and interpretation of the fluorescence trends. Reported studies of the maturation times of mTagBFP2, mEmerald, CyOFP1 and mCardinal2 were in the range of 10–30 min [32, 46–48], whereas fluorescent protein stability in *Escherichia coli* cells can last up to several hours [49]. In this regard, in vivo half-life times of the here used reporter proteins need to be determined. To better represent the expressed cellular physiologies in response to dynamic environmental changes, corresponding markers with rather low half-life times are preferred. Consequently, reporter strains can be enhanced by the integration of destabilized variants of the fluorescent proteins, which are prolonged with degradation tags to allow a faster decay of evolved fluorescent signals by the native cellular degradation system and thus reduce the half-life times [9].

### Evaluation of single-cell fluorescence and potential occurrence of population heterogeneity

The evaluation of population heterogeneity in bioprocesses can rely on different parameters. While higher mean-to-median ratios indicate the presence of a bimodal distribution, the coefficient of variance is rather used to detect broadly distributed histograms [35]. However, both parameters have their limitations [13, 50], which was also observed in this study. The MMR values were effective to reveal bimodal distributions as long as the subpopulations were clearly separated. In cases of closely adjacent subpopulations, the MMR values struggled to reflect this. In contrary, CV values were advantageous to highlight broad distributions, but failed to detect bimodal histograms. In any case, the visuals of histograms should always be included for the evaluation, as well. According to these parameters, population heterogeneity was observed under both homogeneous conditions in the stirred-tank bioreactor, which was already reported for the *E. coli* triple reporter strain [13], and during the two-compartment bioreactor cultivation with dynamic conditions. Notably, differences in cellular phenotypes were found particularly for the general stress response and the product formation at the beginning and the end of the L-phenylalanine production process. The *rpoS* gene seems vulnerable to polymorphism, meaning that this genetic segment often differs in cells of a microbial population originating from an isogenic culture. Deviations of genotypes were followed by a variety of phenotypes, which led to altered functions of the corresponding sigma factor and thus, probably to differences in expression levels of the related mTagBFP2 marker [51, 52]. Interestingly, cells with higher blue fluorescence at the end of the process simultaneously expressed higher green fluorescence, so there might be a strong connection between the general stress response and cell growth (Additional file 1: Fig. S2). Conversely, the occurrence of subpopulations for the product formation marker could be related to the absolute plasmid copy number in the cells, which tends to vary based on the prevailing bioprocess conditions and the cellular state [53]. Hence, cells with a higher number of plasmids potentially express higher red fluorescence and vice versa. In the end, it is worth mentioning that subpopulations during the product formation phase in both processes particularly emerged after the depletion of L-tyrosine, indicating an important role of this amino acid for the emergence of population heterogeneity of this strain.

In summary, population heterogeneity may be present in bioprocesses even under homogeneous conditions. Besides the identification of possible sources for this phenomenon, it would be interesting to further analyze distinct subpopulations and investigate the stability of the expressed cellular phenotypes. This could be realized by sorting of subpopulations during bioprocesses with a fluorescence-activated cell sorter and subsequent cultivation of sorted populations in distinct environments under the same conditions. Furthermore, extended studies on isolated subpopulations derived from an isogenic culture could be analyzed with omics technologies to elucidate underlying sources and concrete differences.

## Conclusions

The repetitive but short exposure of L-phenylalanine producing *Escherichia coli* cells in a novel two-compartment bioreactor did not affect the product formation, but reduced the cell growth compared to a stirred-tank bioreactor cultivation. Nevertheless, mean-to-median ratios, coefficient of variances and histograms of the expressed fluorescent proteins revealed the prevalence of population heterogeneity in both processes, particularly for the general stress response and the product formation of single cells at the process start and ends. These findings confirm the omnipresence of population heterogeneity in bioprocesses and emphasize the supportive role of reporter strains to better understand and identify potential differences of cellular characteristics during bioprocesses. Although more data is mandatory to comprehend this complex phenomenon in detail, reporter strains could be a major step to facilitate future scale-up procedures and to enable better control of industrial-scale bioprocesses.

### Supplementary Information

**Additional file 1: Table S1.** Derivatization method for the pre-treatment of samples for quantification of L-phenylalanine and L-tyrosine with HPLC. **Table S2.** Gradient profile applied during HPLC measurement of derivatized samples for quantification of L-phenylalanine and L-tyrosine. Mobile phase A is a monosodium phosphate solution (20 mM, pH 7.6 adjusted with sodium hydroxide), whereas mobile phase B is a mixture of 45% acetonitrile, 45% methanol and 10% de-ionized water. **Figure S1.** Concentration of L-tyrosine during L-phenylalanine production in the stirred-tank reactor (STR ●) or two-compartment bioreactor (TCB ▲) with an *Escherichia coli* quadruple reporter strain (4RP). Vertical lines separate the phases. **Table S3.** Integral carbon balance of the L-phenylalanine production in a stirred-tank bioreactor with *Escherichia coli* 4RP. Times are normalized to the start of the biomass production phase and the product formation phase. Other products include citrate, ethanol, malate, succinate, L-glutamate, L-tyrosine and L-tryptophane (L-phe—L-phenylalanine). **Table S4.** Integral carbon balance of the L-phenylalanine production in the two-compartment bioreactor with *Escherichia coli* 4RP. Times are normalized to the start of the biomass production phase and the product formation phase. Other products include citrate, ethanol, malate, succinate, L-glutamate, L-tyrosine and L-tryptophane (L-phe—L-phenylalanine). **Table S5.** Mean-to-median ratio and the coefficient of variance of the expressed fluorescence intensities at 448/45 nm (general stress response), 527/32 nm (growth behavior), 586/42 nm (oxygen limitation) and 660/10 nm (product formation) during the L-phenylalanine production process in a stirred-tank bioreactor with *Escherichia coli* 4RP. **Table S6.** Mean-to-median ratio and the coefficient of variance of the expressed fluorescence intensities at 448/45 nm (general stress response), 527/32 nm (growth behavior), 586/42 nm (oxygen limitation) and 660/10 nm (product formation) during the L-phenylalanine production process in a two-compartment bioreactor with *Escherichia coli* 4RP. Each time stamp included the measurements of three technical replicates. **Figure S2.** Density plot of cells from the L-phenylalanine production in the two-compartment bioreactor at the end of the process. This plot correlates the expressed fluorescence intensities at 448/45 nm to 527/32 nm.

## Data Availability

The datasets used and/or analysed during the current study are available from the corresponding author on reasonable request.
