# Supplementary material for: Impact of mixing insufficiencies on L-phenylalanine production with an Escherichia coli reporter strain in a novel two-compartment bioreactor
Source: Microb Cell Fact. 2023 Aug 13;22:153. doi: 10.1186/s12934-023-02165-4 (PMC10424407; doi:10.1186/s12934-023-02165-4)
Supplement: Supplementary file 1 — Additional file 1: Table S1. Derivatization method for the pre-treatment of samples for quantification of L-phenylalanine and L-tyrosine with HPLC. Table S2. Gradient profile applied during HPLC measurement of derivatized samples for quantification of L-phenylalanine and L-tyrosine. Mobile phase A is a monosodium phosphate solution (20 mM, pH 7.6 adjusted with sodium hydroxide), whereas mobile phase B is a mixture of 45% acetonitrile, 45% methanol and 10% de-ionized water. Figure S1. Concentration of L-tyrosine during L-phenylalanine production in the stirred-tank reactor (STR ●) or two-compartment bioreactor (TCB ▲) with an Escherichia coli quadruple reporter strain (4RP). Vertical lines separate the phases. Table S3. Integral carbon balance of the L-phenylalanine production in a stirred-tank bioreactor with Escherichia coli 4RP. Times are normalized to the start of the biomass production phase and the product formation phase. Other products include citrate, ethanol, malate, succinate, L-glutamate, L-tyrosine and L-tryptophane (L-phe—L-phenylalanine). Table S4. Integral carbon balance of the L-phenylalanine production in the two-compartment bioreactor with Escherichia coli 4RP. Times are normalized to the start of the biomass production phase and the product formation phase. Other products include citrate, ethanol, malate, succinate, L-glutamate, L-tyrosine and L-tryptophane (L-phe—L-phenylalanine). Table S5. Mean-to-median ratio and the coefficient of variance of the expressed fluorescence intensities at 448/45 nm (general stress response), 527/32 nm (growth behavior), 586/42 nm (oxygen limitation) and 660/10 nm (product formation) during the L-phenylalanine production process in a stirred-tank bioreactor with Escherichia coli 4RP. Table S6. Mean-to-median ratio and the coefficient of variance of the expressed fluorescence intensities at 448/45 nm (general stress response), 527/32 nm (growth behavior), 586/42 nm (oxygen limitation) and 660/10 nm (product formation [file 12934_2023_2165_MOESM1_ESM.docx]

Additional file 1

**Impact of mixing insufficiencies on L-phenylalanine production with an *Escherichia coli* reporter strain in a novel two-compartment bioreactor**

Manh Dat Hoang ^1^, Ingmar Polte ^1^, Lukas Frantzmann ^1^, Nikolas von den Eichen ^1^, Anna-Lena Heins ^1, *^, Dirk Weuster-Botz ^1^

^1^ Technical University of Munich, School of Engineering and Design, Chair of Biochemical Engineering, Boltzmannstr. 15, 85748 Garching, Germany

^*^ Corresponding author: anna-lena.heins@tum.de

Table S1. Derivatization method for the pre-treatment of samples for quantification of L-phenylalanine and L-tyrosine with HPLC.

| **Step** | **Description** |
| --- | --- |
| 1 | Transfer of 10 µL of sample to the destination vial filled with 650 µL 0.04 M bicine buffer and 8 µL of a diluted mercaptopropionic acid solution (2.5 µL in 1 mL 0.04 bicine buffer) |
| 2 | Mixing and resuspension for three times |
| 3 | Incubation for 30 s |
| 4 | Addition of 20 µL of the 1:10 diluted iodoacetic acid solution to destination vial |
| 5 | Mixing and resuspension for three times |
| 6 | Incubation for 30 s |
| 7 | Addition of 70 µL of the 1:7 diluted ortho-phtaldialdehyde solution to destination vial |
| 8 | Mixing and resuspension for three times |
| 9 | Injection of 20 µL of derivatized sample into the HPLC |

Table S2. Gradient profile applied during HPLC measurement of derivatized samples for quantification of L-phenylalanine and L-tyrosine. Mobile phase A is a monosodium phosphate solution (20 mM, pH 7.6 adjusted with sodium hydroxide), whereas mobile phase B is a mixture of 45% acetonitrile, 45% methanol and 10% de-ionized water.

| **Time, min** | **Flow rate (mL min^-1^)** | **Mobile phase A, %** | **Mobile phase B, %** |
| --- | --- | --- | --- |
| 0.00 | 1.0 | 100 | 0 |
| 3.00 | 1.0 | 100 | 0 |
| 8.50 | 1.0 | 75 | 25 |
| 28.50 | 1.0 | 60 | 40 |
| 30.00 | 1.0 | 60 | 40 |
| 30.02 | 1.0 | 0 | 100 |
| 32.00 | 1.0 | 0 | 100 |
| 34.00 | 1.2 | 20 | 80 |
| 38.00 | 1.0 | 100 | 0 |
| 43.00 | 1.0 | 100 | 0 |
| 43.02 | 1.0 | 100 | 0 |

Figure S1. Concentration of L-tyrosine during L-phenylalanine production in the stirred-tank reactor (STR ●) or two-compartment bioreactor (TCB ▲) with an *Escherichia coli* quadruple reporter strain (4RP). Vertical lines separate the phases.

Table S3. Integral carbon balance of the L-phenylalanine production in a stirred-tank bioreactor with *Escherichia coli* 4RP. Times are normalized to the start of the biomass production phase and the product formation phase. Other products include citrate, ethanol, malate, succinate, L-glutamate, L-tyrosine and L-tryptophane (L-phe – L-phenylalanine).

| **Time, h** | **Biomass, mol%** | **Glycerol, mol%** | **Acetate, mol%** | **L-phe, mol%** | **Carbon dioxide, mol%** | **Others, mol%** |
| --- | --- | --- | --- | --- | --- | --- |
| 0 | 5.4 | 71.7 | 1.0 | 2.6 | 0.0 | 19.2 |
| 14.0 | 29.9 | 16.7 | 1.0 | 1.8 | 29.1 | 19.6 |
| 15.3 | 42.0 | 0.6 | 1.0 | 1.5 | 36.4 | 19.4 |
| 18.3 | 43.7 | 0.4 | 0.7 | 1.0 | 38.0 | 14.1 |
| 21.3 | 40.8 | 0.2 | 0.5 | 1.3 | 39.6 | 11.3 |
| 24.7 | 43.3 | 0.2 | 0.3 | 1.4 | 42.0 | 8.8 |
| 27.9 | 41.2 | 0.2 | 0.2 | 0.7 | 48.6 | 6.3 |
| 38.0 | 37.0 | 0.1 | 0.2 | 0.2 | 61.5 | 2.5 |
| 39.9 | 35.1 | 0.1 | 0.1 | 0.2 | 64.8 | 3.2 |
| 40.9 | 34.3 | 0.1 | 0.1 | 0.3 | 66.2 | 3.3 |
| 43.3 | 34.0 | 0.1 | 0.1 | 0.8 | 67.5 | 2.9 |
| 45.9 | 32.1 | 0.1 | 0.1 | 1.7 | 68.0 | 2.6 |
| 51.9 | 29.1 | 0.1 | 0.1 | 6.3 | 67.7 | 2.3 |
| 62.1 | 23.7 | 0.1 | 0.1 | 13.4 | 67.0 | 2.0 |
| 64.9 | 22.6 | 0.1 | 0.1 | 13.5 | 66.9 | 2.1 |
| 67.9 | 21.1 | 0.1 | 0.1 | 15.4 | 67.0 | 2.2 |
| 70.9 | 19.7 | 0.1 | 0.1 | 14.4 | 67.2 | 2.5 |
| 75.3 | 18.5 | 0.1 | 0.4 | 14.9 | 67.5 | 2.8 |
| 87.3 | 15.9 | 0.2 | 3.4 | 13.8 | 67.4 | 2.9 |
| 89.1 | 15 | 0.3 | 4.5 | 13.5 | 66.8 | 3.4 |
| 89.9 | 14.9 | 0.4 | 4.9 | 13.0 | 66.6 | 3.4 |

Table S4. Integral carbon balance of the L-phenylalanine production in the two-compartment bioreactor with *Escherichia coli* 4RP. Times are normalized to the start of the biomass production phase and the product formation phase. Other products include citrate, ethanol, malate, succinate, L-glutamate, L-tyrosine and L-tryptophane (L-phe – L-phenylalanine).

| **Time, h** | **Biomass, mol%** | **Glycerol, mol%** | **Acetate, mol%** | **L-phe, mol%** | **Carbon dioxide, mol%** | **Others, mol%** |
| --- | --- | --- | --- | --- | --- | --- |
| 0 | 1.2 | 75.8 | 0.5 | 3.2 | 0 | 19.3 |
| 12.0 | 16.8 | 34.4 | 0.5 | 3.8 | 21.8 | 19.4 |
| 13.7 | 28.2 | 11.6 | 0.5 | 4.1 | 33.6 | 19.6 |
| 14.6 | 38.0 | 0.3 | 0.5 | 4.1 | 41.0 | 19.8 |
| 17.6 | 36.5 | 0.2 | 0.4 | 4.1 | 45.2 | 15.4 |
| 20.6 | 39.4 | 0.2 | 0.3 | 4.2 | 49.2 | 12.2 |
| 23.6 | 40.1 | 0.2 | 0.2 | 4.3 | 53.3 | 10.6 |
| 25.4 | 38.1 | 0.1 | 0.3 | 3.8 | 54.5 | 9.6 |
| 34.7 | 38.3 | 0.1 | 0.1 | 2.3 | 63..0 | 3.9 |
| 36.7 | 38.6 | 0.1 | 0.1 | 2.0 | 64.8 | 3.5 |
| 37.8 | 37.7 | 0.1 | 0.1 | 2.0 | 66.1 | 5.3 |
| 38.8 | 37.3 | 0.1 | 0.1 | 2.1 | 66.4 | 5.2 |
| 40.8 | 36.5 | 0 | 0.1 | 2.2 | 66.3 | 4.8 |
| 43.8 | 34.0 | 0 | 0.1 | 3.7 | 67.1 | 4.2 |
| 45.3 | 33.6 | 0 | 0.1 | 4.9 | 66.7 | 4.0 |
| 59.4 | 25.9 | 0 | 0.1 | 15.4 | 66.5 | 2.7 |
| 62.4 | 24.6 | 0 | 0.1 | 16.7 | 66.8 | 2.9 |
| 65.4 | 23.1 | 0 | 0.1 | 17.1 | 67.2 | 3.1 |
| 68.4 | 22.4 | 0.1 | 0.1 | 18.3 | 67.5 | 3.1 |
| 70.4 | 21.6 | 0.1 | 0.2 | 18.5 | 67.8 | 3.4 |
| 75.4 | 19.3 | 0.1 | 0.3 | 18.7 | 68.6 | 3.7 |
| 84.1 | 17.1 | 0.1 | 1.2 | 19.4 | 69.2 | 3.6 |
| 87.1 | 17.6 | 0.1 | 1.9 | 18.2 | 68.8 | 4.5 |
| 90.1 | 16.7 | 0.1 | 2.8 | 17.7 | 68.1 | 5.3 |
| 92.1 | 16.1 | 0.1 | 3.4 | 16.7 | 67.6 | 6.0 |
| 94.1 | 15.9 | 0.1 | 4.2 | 16.4 | 66.9 | 6.6 |

Table S5. Mean-to-median ratio and the coefficient of variance of the expressed fluorescence intensities at 448/45 nm (general stress response), 527/32 nm (growth behavior), 586/42 nm (oxygen limitation) and 660/10 nm (product formation) during the L-phenylalanine production process in a stirred-tank bioreactor with *Escherichia coli* 4RP.

| **Time, h** | **448/45 nm**  **General stress response** | | **527/32 nm  Growth behavior** | | **586/42 nm**  **Oxygen limitation** | | **660/10 nm**  **Product formation** | |
| --- | --- | --- | --- | --- | --- | --- | --- | --- |
|  | **Mean/Median, -** | **CV, %** | **Mean/Median, -** | **CV, %** | **Mean/Median, -** | **CV, %** | **Mean/Median, -** | **CV, %** |
| 14.0 | 1.61 | 2.01 | 1.35 | 1.22 | 1.49 | 1.73 | 2.74 | 2.14 |
| 15.3 | 1.68 | 2.21 | 1.37 | 1.44 | 1.54 | 2.22 | 2.92 | 2.09 |
| 18.3 | 1.30 | 1.21 | 1.30 | 1.12 | 1.32 | 1.49 | 2.27 | 1.92 |
| 21.3 | 1.29 | 1.55 | 1.37 | 3.13 | 1.32 | 1.54 | 2.39 | 1.87 |
| 24.7 | 1.19 | 1.17 | 1.28 | 1.18 | 1.24 | 1.23 | 2.02 | 1.44 |
| 27.9 | 1.15 | 1.00 | 1.23 | 1.02 | 1.22 | 1.12 | 2.02 | 1.37 |
| 38.0 | 1.11 | 0.84 | 1.18 | 0.90 | 1.19 | 1.06 | 1.89 | 1.14 |
| 39.9 | 1.08 | 0.71 | 1.15 | 0.81 | 1.18 | 0.97 | 1.68 | 1.02 |
| 40.9 | 1.11 | 1.13 | 1.17 | 1.24 | 1.23 | 1.83 | 1.68 | 1.10 |
| 43.3 | 1.08 | 0.63 | 1.14 | 0.71 | 1.18 | 1.10 | 1.41 | 1.00 |
| 45.9 | 1.09 | 0.66 | 1.12 | 0.62 | 1.13 | 0.87 | 1.19 | 0.77 |
| 51.9 | 1.32 | 0.88 | 1.10 | 0.61 | 1.12 | 0.96 | 1.05 | 0.61 |
| 62.1 | 1.49 | 1.34 | 1.10 | 1.16 | 1.13 | 1.69 | 1.07 | 0.67 |
| 64.9 | 1.44 | 0.88 | 1.12 | 0.95 | 1.11 | 1.60 | 1.10 | 0.64 |
| 67.9 | 1.50 | 1.08 | 1.13 | 2.65 | 1.11 | 0.94 | 1.11 | 0.83 |
| 70.9 | 1.49 | 0.86 | 1.12 | 0.72 | 1.11 | 1.33 | 1.12 | 0.63 |
| 75.3 | 1.52 | 0.94 | 1.14 | 0.87 | 1.15 | 1.65 | 1.16 | 0.65 |
| 87.3 | 1.77 | 0.94 | 1.13 | 0.61 | 1.18 | 1.08 | 1.11 | 0.64 |
| 89.1 | 1.68 | 0.92 | 1.14 | 0.64 | 1.23 | 1.17 | 1.13 | 0.66 |
| 89.9 | 1.72 | 0.92 | 1.13 | 0.65 | 1.24 | 1.25 | 1.12 | 0.66 |

Table S6. Mean-to-median ratio and the coefficient of variance of the expressed fluorescence intensities at 448/45 nm (general stress response), 527/32 nm (growth behavior), 586/42 nm (oxygen limitation) and 660/10 nm (product formation) during the L-phenylalanine production process in a two-compartment bioreactor with *Escherichia coli* 4RP. Each time stamp included the measurements of three technical replicates.

| **Time, h** | **448/45 nm**  **General stress response** | | **527/32 nm  Growth behavior** | | **586/42 nm**  **Oxygen limitation** | | **660/10 nm**  **Product formation** | |
| --- | --- | --- | --- | --- | --- | --- | --- | --- |
|  | **Mean/Median, -** | **CV, %** | **Mean/Median, -** | **CV, %** | **Mean/Median, -** | **CV, %** | **Mean/Median, -** | **CV, %** |
| 12.0 | 1.23 | 1.24 | 1.19 | 1.18 | 1.19 | 0.84 | 1.55 | 1.11 |
| 13.7 | 1.25 | 0.86 | 1.19 | 0.71 | 1.18 | 0.81 | 1.47 | 1.04 |
| 14.6 | 1.26 | 0.86 | 1.19 | 0.71 | 1.18 | 0.83 | 1.48 | 1.06 |
| 17.6 | 1.28 | 0.85 | 1.21 | 0.71 | 1.20 | 0.81 | 1.43 | 0.99 |
| 20.6 | 1.32 | 1.27 | 1.22 | 1.03 | 1.20 | 1.12 | 1.49 | 1.09 |
| 23.6 | 1.27 | 0.91 | 1.24 | 0.71 | 1.21 | 0.79 | 1.52 | 1.01 |
| 25.4 | 1.30 | 0.92 | 1.25 | 0.73 | 1.23 | 0.80 | 1.42 | 0.91 |
| 34.7 | 1.40 | 1.15 | 1.28 | 1.34 | 1.25 | 0.83 | 1.48 | 0.96 |
| 36.7 | 1.36 | 1.01 | 1.31 | 0.86 | 1.30 | 0.92 | 1.63 | 1.05 |
| 37.8 | 1.37 | 1.03 | 1.29 | 0.83 | 1.27 | 0.85 | 1.53 | 1.00 |
| 38.8 | 1.33 | 1.09 | 1.28 | 0.81 | 1.27 | 0.89 | 1.55 | 1.00 |
| 40.8 | 1.30 | 0.98 | 1.28 | 0.85 | 1.27 | 0.97 | 1.51 | 0.98 |
| 43.8 | 1.31 | 0.98 | 1.26 | 0.81 | 1.25 | 0.83 | 1.39 | 0.92 |
| 45.3 | 1.33 | 1.00 | 1.25 | 0.84 | 1.25 | 0.84 | 1.36 | 0.89 |
| 59.4 | 1.50 | 1.07 | 1.28 | 0.85 | 1.30 | 0.98 | 1.31 | 0.84 |
| 62.4 | 1.71 | 1.20 | 1.39 | 0.91 | 1.37 | 1.05 | 1.40 | 0.91 |
| 65.4 | 1.80 | 1.26 | 1.45 | 0.93 | 1.42 | 1.12 | 1.42 | 0.92 |
| 68.4 | 1.85 | 1.31 | 1.44 | 0.92 | 1.41 | 1.14 | 1.42 | 0.92 |
| 70.4 | 1.83 | 1.32 | 1.45 | 0.95 | 1.43 | 1.19 | 1.41 | 0.91 |
| 75.4 | 1.72 | 1.20 | 1.35 | 0.89 | 1.42 | 1.18 | 1.33 | 0.83 |
| 84.1 | 1.96 | 1.35 | 1.45 | 0.95 | 1.54 | 1.33 | 1.36 | 0.87 |
| 87.1 | 2.04 | 1.42 | 1.45 | 0.90 | 1.56 | 1.28 | 1.34 | 0.85 |
| 90.1 | 2.11 | 1.44 | 1.43 | 0.88 | 1.56 | 1.23 | 1.33 | 0.83 |
| 92.1 | 2.11 | 1.40 | 1.41 | 0.85 | 1.55 | 1.17 | 1.31 | 0.82 |
| 94.1 | 2.16 | 1.44 | 1.40 | 0.83 | 1.57 | 1.21 | 1.31 | 0.81 |

Figure S2. Density plot of cells from the L-phenylalanine production in the two-compartment bioreactor at the end of the process. This plot correlates the expressed fluorescence intensities at 448/45 nm to 527/32 nm.
